# Isolation-protocol, characterization, and *in-vitro* performance of equine umbilical vein endothelial cells

**DOI:** 10.3389/fvets.2024.1421946

**Published:** 2024-10-01

**Authors:** Ulrike Lessiak, Maria Melchert, Ingrid Walter, Stefan Kummer, Barbara Nell, Waltraud Tschulenk, Barbara Pratscher

**Affiliations:** ^1^Ophthalmology Unit, Department of Companion Animals and Horses, University of Veterinary Medicine Vienna, Vienna, Austria; ^2^Centre for Animal Reproduction, Department of Companion Animals and Horses, University of Veterinary Medicine Vienna, Vienna, Austria; ^3^Department of Biomedical Science and Pathobiology, University of Veterinary Medicine Vienna, Vienna, Austria; ^4^VetCore Facility for Research, University of Veterinary Medicine Vienna, Vienna, Austria; ^5^Research Unit Internal Medicine, Department of Companion Animals and Horses, University of Veterinary Medicine Vienna, Vienna, Austria

**Keywords:** EqUVEC, angiogenesis, equine endothelial cells, immunohistology, TEM, cell culture

## Abstract

Angiogenesis plays a crucial role in various physiological and pathological conditions. However, research in equine angiogenesis is relative limited, necessitating the development of suitable *in-vitro* models. To effectively analyze angiogenesis in-vitro, it is essential to target the specific cells responsible for this process, namely endothelial cells. Human umbilical vein endothelial cells (HUVECs) are one of the most used *in vitro* models for studying angiogenesis in humans. Serving as an equivalent to HUVECs, we present a comprehensive isolation protocol for equine umbilical vein endothelial cells (EqUVECs) with relatively minimal requirements, thereby enhancing accessibility for researchers. Umbilical cords obtained from five foals were used to isolate endothelial cells, followed by morphological and immunohistochemical identification. Performance of the cells in various assays commonly used in angiogenesis research was studied. Additionally, EqUVEC expression of vascular endothelial growth factor (VEGF) was assessed using ELISA. EqUVECs exhibited endothelial characteristics, forming a homogeneous monolayer with distinctive morphology. Immunohistochemical staining confirmed positive expression of key endothelial markers including von Willebrand factor (vWF), CD31, and vascular endothelial growth factor receptor-2 (VEGFR-2). Furthermore, performance assessments in *in-vitro* assays demonstrated the viability, proliferation, migration, tube formation and VEGF-expression capabilities of EqUVECs. The findings suggest that EqUVECs are a promising *in-vitro* model for studying equine angiogenesis, offering a foundation for further investigations into equine-specific vascular processes and therapeutic interventions.

## 1 Introduction

Angiogenesis, the formation of new blood vessels from pre-existing ones, plays a pivotal role in various physiological and pathological conditions (1). The regulation through a balance of pro-and anti-angiogenic factors is essential for maintaining a healthy and well-functioning tissue (2–4). Dysregulation of angiogenesis can lead to numerous diseases in humans, including ocular neovascularization and wound healing disorders, eventually causing tissue and organ dysfunctions (5–7). Pathological vascularization is one of the factors considered responsible for metastasis and cancer development, making it a key area within recent human cancer research (6, 8).

In horses, dysregulated angiogenesis is associated with various conditions, such as laminitis and ocular pathologies, including equine recurrent uveitis (ERU) (9–16). ERU is characterized by recurrent episodes of inflammation within the eye, leading to vision impairment and blindness (17). Abnormal growth of blood vessels within the uveal tract contributes to the progression of this condition, exacerbating tissue damage and inflammation (17). Furthermore, tumors like sarcoids and squamous cell carcinomas in horses May also exhibit aberrant angiogenic processes (12, 18, 19). Additionally, impaired wound healing is linked to dysregulated angiogenesis (9, 15, 16). Inadequate blood supply to the wound site hinders the delivery of essential nutrients and oxygen, delaying the healing process.

Despite the clinical relevance, research about the topic of angiogenesis as well as pro-and anti-angiogenic treatments for horses is relatively limited (7). Considering the various functions of the vascular system, it is vital to understand the molecular mechanisms of angiogenesis to develop therapeutic approaches. *In-vitro* models provide a valuable tool for investigating angiogenesis by offering controlled environments for studying molecular mechanisms and assessing potential therapeutic interventions and species-specific responses. These models bridge the gap between *in-vivo* studies and clinical applications (20, 21).

Endothelial cells (ECs) are primarily responsible for physiological and pathological angiogenesis and are thus extensively used in angiogenesis research. Various EC models have been studied, using sources such as pulmonary arteries, digital or jugular veins (22–25). However, the endothelium is highly heterogeneous, with arterial ECs significantly differing from venous ECs in morphological and functional properties (26–28). Notably, results obtained from human umbilical vein endothelial cells (HUVECs) can be extended to other EC types and seem to reflect the behavior of arterial ECs as well (29). Furthermore, umbilical cords are considered medical waste and are therefore easily accessible (29). The HUVEC model, specifically, has proven beneficial in studying both physiological and pathological effects in response to various stimuli and pathways, either in isolation or in co-culture with other cell types (30). *In-vitro* HUVEC models have found to be useful for studying various aspects of endothelial function, including monocyte adhesion, endothelial damage, and repair (31). The HUVEC model allows for exposure of the cells to shear stress under controlled flow conditions, mimicking *in-vivo* blood flow (32). Furthermore, this model is used for assessing the impact of novel drugs on human endothelium, contributing to the understanding of diverse biological processes and diseases such as inflammation, cancer, and diabetes mellitus (33–37).

Considering this, EqUVECs, serving as an equivalent to HUVECs, could enhance our understanding of equine angiogenesis. This research May help develop effective therapeutic strategies for pro-and anti-angiogenic treatments in horses. Currently, there is no equine-umbilical-vein-endothelial-cell-line commercially available; thus, we aimed to describe a method for isolating EqUVECs with relatively minimal equipment in order to increase accessibility of EqUVECs for researchers.

## 2 Materials and equipment

The materials and equipment required for umbilical cord sampling, cell isolation and processing, and immunolabeling are listed in Tables 1–4.

**Table 1:** Solutions used and preparation of EqUVEC transport medium.

Component	Source	Final concentration
Colistin	Sigma-Aldrich, Saint Louis, Missouri, United States	10 μg/mL
Vancomycin	Sigma-Aldrich, Saint Louis, Missouri, United States	10 μg/mL
Penicillin (10.000 units/mL)-Streptomycin (10.000 μg/mL) (100x)	Life Technologies, Thermofisher Science, Carlsbad, California, United States	1x
PBS	Sigma-Aldrich, Saint Louis, Missouri, United States	To final volume of 500 mL

**Table 2:** Solutions used and preparation of EqUVEC culture medium.

Component	Source	Volume added for 500 mL	Final concentration
FCS	Life Technologies, Thermofisher Science, Carlsbad, California, United States	100 mL	20%
HEPES 1 M	Life Technologies, Thermofisher Science, Carlsbad, California, United States	15 mL	15 mM
Antibiotic (10.000 units/mL penicillin) - Antimycotic (10,000 μg/mL streptomycin, 25 μg/mL Amphotericin B) (100x)	Life Technologies, Thermofisher Science, Carlsbad, California, United States	20 mL	1x
DMEM + GlutaMAX ™	Life Technologies, Thermofisher Science, Carlsbad, California, United States	To final volume of 500 mL	-

**Table 3:** Equipment used for isolation.

Equipment
Hood for cell culture with vertical laminar flow and equipped with UV light for decontamination
Water-bath with temperature control
Centrifuge
Incubator with temperature and gas composition controls
Optical microscope
Equipment for material sterilization
Stainless steel bowl
Hemostats (2x)
Scissor
Forceps (2x)
Cannulae 23G
Syringes 50 mL, 30 mL, 10 mL
Cotton gauze (autoclaved)
Gloves
Tubes 50 mL

**Table 4:** Primary antibodies used for immunolabeling.

Primary antibody	Clone	Dilution	Pre-treatment	Source
van Willebrand Factor (vWF)	Polyclonal rabbit	1:7000	1 mg/mL Protease from *Streptomyces griseus*	Agilent Dako, Santa Clara, CA
CD31	Polyclonal rabbit	1:500	Boil in Tris-EDTA (pH 9) for 30 min (steamer)	Cell Marque Corporation, Rocklin, CA
Vascular Endothelial Growth Factor Receptor-2 (VEGFR-2)	Monoclonal mouse	1:200	Boil in citrate buffer (pH 6) for 30 min (steamer)	Santa Cruz Biotechnology, Dallas, TX

### 2.1 Animals

Umbilical cords of foals containing two umbilical arteries (UA) and one vein (UV) were harvested and included in the study after delivery (Table 5). Informed owner consent was obtained from all owners at the time of hospitalization of the horses (Mare 1, 2, 4) whose umbilical veins were harvested after placental shedding and involved in the study. The collection of umbilical veins from research animals (Mare 3 and 5) was approved by the competent authority for animal experimentation in Austria (Federal Ministry for Science and Research, license number 2020–0.547.889). The mares and foals included in the study were healthy, the deliveries unassisted or required minor assistance.

**Table 5:** List of mares the samples were obtained from.

Mare	Breed	Age (years)	Weight a.p.* (kg)	Weight p.p.** (kg)	Placenta	Umbilical vein	Foaling/foal	Gestation (days)
Horse 1 / EqUVEC 1	Pony	11	315	274	Unremarkable	Unremarkable	Unassisted foaling	329
Horse 2 / EqUVEC 2	Warmblood	16	676	610	Unremarkable	Unremarkable	Assisted foaling	369
Horse 3 / EqUVEC 3	Shetland-Pony	9	280.5	253	Unremarkable	Unremarkable	Assisted foaling (malposture)	326
Horse 4 / EqUVEC 4	Thoroughbred	21	-	480	Unremarkable	Unremarkable	Assisted foaling (dystocia due to malposition)	-
Horse 5 / EqUVEC 5	Shetland-Pony	16	214	180	Unremarkable	Unremarkable	Unassisted foaling	313

*a.p. = ante partum. **p.p. = post partum.

## 3 Methods

### 3.1 Sampling

After harvesting, umbilical cords were carefully cleaned and rinsed with isotonic saline solution 0.9% (B. Braun, Melsungen, Germany) to eliminate any remaining blood residues. Samples were then transferred to a transport flask containing 500 mL PBS supplemented with antibiotics (10 μg/mL colistin, 10 μg/mL vancomycin, 100 units/mL of penicillin and 100 μg/mL of streptomycin) and stored at 4° until further processing (Table 1). Endothelial cells were isolated within 12 h after harvesting.

### 3.2 Isolation and cell culture of endothelial cells

**Figure 1:**
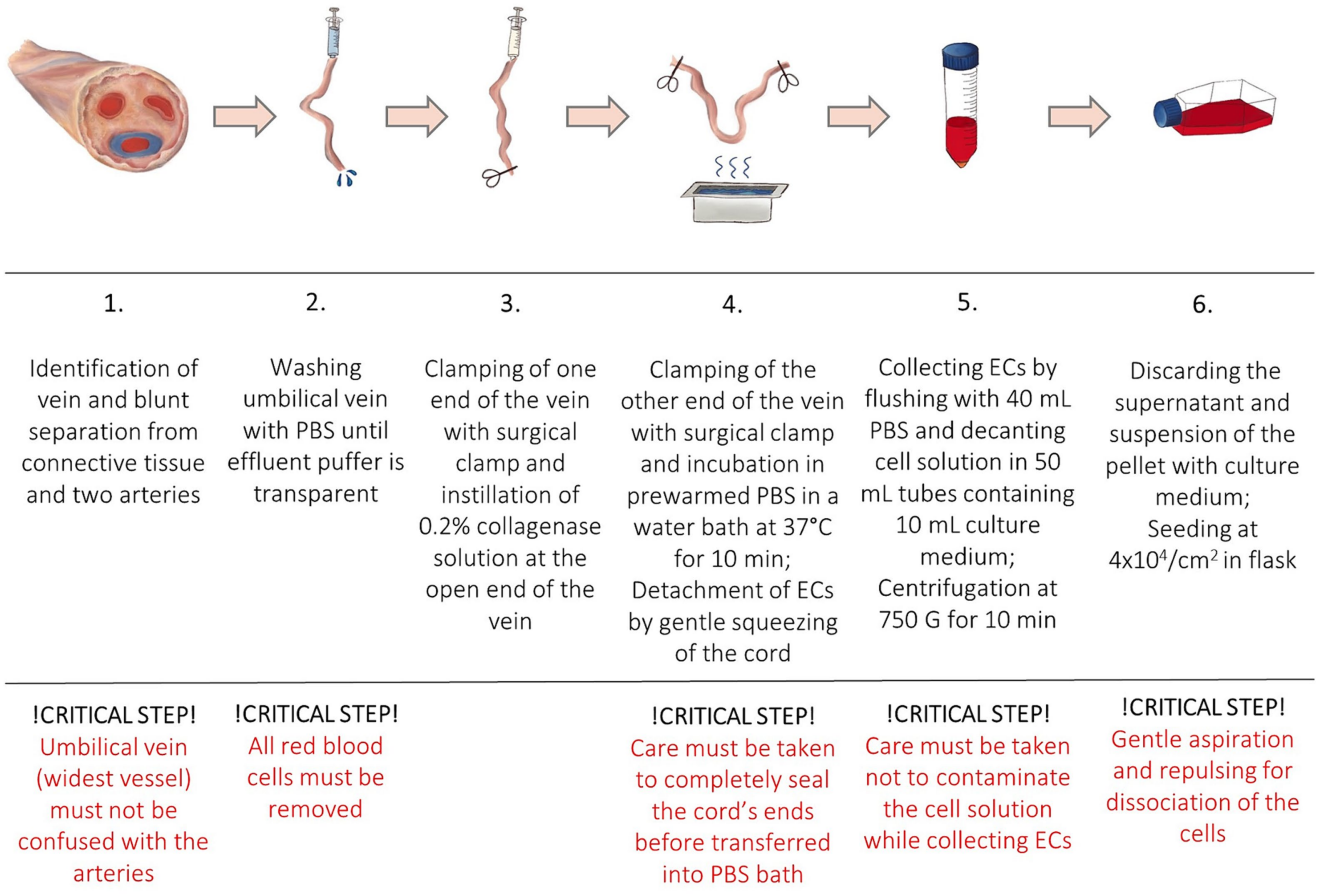
Step-by-Step isolation protocol and critical steps.

The protocol was adapted from a human endothelial vein cell (HUVEC) isolation protocol by Baudin et al. (30). Isolated cells were used in a drug-efficiency experiment (38). All samples were processed in a laminar flow system under sterile conditions. After removal of the connective tissue and umbilical arteries, the umbilical vein was again rinsed with 50 mL PBS to remove the red blood cells until transparent buffer was effluent. One end of the vein was tightly clamped using a sterile surgical clamp and filled with prewarmed (37°C) 0.2% collagenase solution to dissociate ECs. The umbilical vein was incubated in a sterile metal dish with prewarmed PBS in a water bath at 37°C. After incubation for 10 min, the cord was gently squeezed to promote further ECs detachment. Dissociated cells were collected by flushing the vein with 40 mL of PBS. The cell solution was decanted into a 50 mL tube containing 10 mL of culture medium. High glucose basis culture medium (DMEM + GlutaMAX ™, Life Technologies, Thermofisher Science, Carlsbad, California, United States) was supplemented with 20% fetal calf serum (FCS), 15 mM HEPES, 100 units/mL of penicillin, 100 μg/mL of streptomycin, and 0.25 μg/mL of Gibco Amphotericin B (Life Technologies, Thermofisher Science, Carlsbad, California, United States) (Table 2). The cell suspension was centrifuged at 450 G for 10 min at room temperature (RT). The supernatant was discarded, and the pellet was resuspended in the complete culture medium. In order to separate cell aggregates, the solution was repeatedly aspirated and repulsed through a 23 G needle (Sterican, 0.6 × 30 mm, Braun, Melsungen, Germany). Cells were seeded at a density of 4 × 10^4^ cells/cm^2^ and incubated at 37°C with 5% CO_2_ concentration and 98% relative humidity. The medium was changed the following day (<24 h) to remove non-endothelial cells with less adhesion capacity. After reaching confluence the monolayer was washed with PBS and trypsinized (1X, Trypsin–EDTA for primary cells, ATCC^®^, USA). After 2–3 min, complete culture medium was added for inactivation of trypsin and the cells were centrifuged. The pellet was resuspended in the complete culture medium, and subcultures were expanded by passaging before being used in further experiments. Cells of passage three were then cryopreserved until further processing and stored at −150°C. EqUVECs up to passage five were used in the assays. To detect cell culture changes cells up to passage 8 were cultured in 6-well plates (Biologix, Delhi, India) and monitored. Each passage was photographed using a 10x phase contrast magnification on a Leica DMi8 inverted microscope (Leica Microsystems GmbH, Wetzlar, Germany). A list of necessary equipment is shown in Table 3; Step-by-Step isolation procedure and critical steps are outlined in Figure 1.

To compare morphological characteristics of equine and human ECs, HUVECs were purchased from American Type Culture Collection (ATCC^®^, USA). Cells were grown in Vascular Basal Medium (ATCC^®^, USA) supplemented with 2% FBS and rhEGF, rhFGF, rhVEGF, rhIGF-1, ascorbic acid, hydrocortisone, heparin and L-glutamine (VEGF Growth kit, ATCC^®^, USA) in a 37°C and 5% CO^2^ humidified incubator. Cells were seeded at 5000 cells/cm^2^ in T-75 flasks. After reaching confluence the monolayer was washed with PBS and trypsinized (1X, Trypsin–EDTA for primary cells, ATCC^®^, USA) before being used in experiments. HUVECs of passage three to five were used in all experiments.

### 3.3 EqUVEC identification and characterization

#### 3.3.1 Morphological characterization of EqUVECs

Transmission electron microscopy (TEM) was used to examine the ultrastructure of EqUVECs. Furthermore, morphological characteristics of EqUVECs were compared to HUVECs. After ECs formed a monolayer, all samples were fixed in 3% buffered glutaraldehyde (pH 7.4, Merck) for 10 min and scraped from the cell culture flask afterwards. Cell culture specimens were pre-embedded in Histogel (Epredia Inc., New Hampshire, USA). After being washed in 0.1 M Soerensen buffer (pH 7.4), the cells were postfixed with 1% osmium tetroxide (Electron Microscopy Sciences, Hatfield, PA, USA) for 2 h at RT. Subsequently, the cells were dehydrated and incubated in increasing ratios of epoxy resin-propylene oxide (1,1, 3:1) and finally pure resin before embedding and polymerization in epoxy resin (Serva, Mannheim, Germany) for 48 h at 60°C. Ultrathin sections of 70 nm were cut, contrasted in methanolic uranyl acetate (Fluka Chemie AG, Buchs, Switzerland) and alkaline lead citrate (Merck KG, Darmstadt, Germany) and examined using an EM 900 electron microscope (Zeiss, Oberkochen, Germany). Digital images were captured using an ImageSP Professional software (SYSPROG, TRS, Moorenweis, Germany).

#### 3.3.2 Identification of EqUVECs using immunohistochemical staining

To confirm the collection of endothelial cells, samples from a representative vessel (positive control) as well as cultured cells were prepared for antibody staining with endothelial markers von Willebrand Factor (vWF), CD31 and vascular endothelial growth factor receptor-2 (VEGFR-2). Endothelial cells were fixed within an 8-well chamber slide (Ibidi, Munich, Germany) in 4% formaldehyde for 10 min. After this the cells were washed twice with PBS for 2 min and dried at room temperature for 15 min and stored at 4°C until further processing. Endogenous peroxidase activity was blocked by incubating the slides in 3% H2O2 in methanol for 15 min. After rinsing, antigen retrieval was performed by heating tissue sections in Tris EDTA buffer at pH 9.0 in a steamer for 45 min. After cooling down for 15 min, a protein block was conducted to prevent non-specific protein binding by incubation with 1.5% goat serum (DAKO, Glostrup, Denmark) in PBS for 30 min. Slides were then incubated with primary antibodies (anti-VEGFR-2, anti-CD31 and anti-vWF) overnight at 4°C in a humidity chamber. To test the specificity of the secondary antibody, a negative control with PBS instead of the primary antibody was used for each staining. Following overnight incubation, sections were rinsed with PBS solution (pH 7.4) and incubated with secondary antibodies (Poly-HRP anti-mouse for VEGFR-2 and Poly-HRP anti-rabbit for CD31 and vWF, all from ImmunoLogic, Duiven, The Netherlands) for 30 min at room temperature in a humidity chamber. After a PBS wash step, immunostaining was visualized using 3,3’ Diaminobenzidine (DAB, Quanto, Richard Allan Scientific, TA-125-QHDX) for 5 min at RT, followed by washing in distilled water and counterstaining with Mayer’s Haemalaun for 3 min before a final rinse in tap water for 10 min. Chambers were assessed independently under light microscopy using a Polyvar microscope (Reichert-Jung, Vienna, Austria). Digital images were captured using a Nikon Ds-Fi1 camera and NIS-Element software (Nikon, Melville, NY, United States). Supplements are listed in Table 4.

### 3.4 Examples of application: performance in different angiogenesis assays

#### 3.4.1 EqUVEC cell viability

The viability of isolated EqUVECs was assessed by measuring their capacity to reduce a substrate, indicative of cellular metabolism. Cell viability was determined using the RealTime-Glo™ MT Cell Viability Assay (Promega Corporation, Fitchburg, WI, United States) according to the manufacturer’s protocol at a 24 h-interval for 96 h. Cells were inoculated at 1.6 × 10^4^ cells/well onto an opaque-walled 96-well plate (Falcon; Becton Dickinson Labware, Plymouth, England). Upon reaching confluence, RealTime-Glo™, MT Cell Viability Substrate and NanoLuc^®^ Enzyme were added. Following a 1-h incubation at 37°C in a 5% CO^2^ humidified chamber, luminescence was quantified using the GloMax^®^ Explorer Multimode Microplate Reader (Promega Corporation, Fitchburg, WI, United States). Each experiment was conducted in triplicate.

#### 3.4.2 EqUVEC cell proliferation

To assess viable cells in proliferation an MTS-assay (3-(4,5-dimethylthiazol-2-yl)-5-(3-carboxymethoxyphenyl)-2-(4-sulfophenyl)-2H-tetrazolium) was performed using the CellTiter 96^®^ AQueous One Solution Cell Proliferation Assay (Promega Corporation, Fitchburg, WI, United States). Briefly, cells were plated (1.6 × 10^4^ cells/well) on 96-well plates in 100 μL/well culture medium with 20 μL/well of CellTiter 96AQueous One Solution (MTS) solution (Promega Corporation, Fitchburg, WI, USA). After incubation at 37°C in a 5% CO^2^ humidified chamber for 1 h, absorbance was measured at 490 nm using the GloMax^®^ Explorer Multimode Microplate Reader (Promega Corporation, Fitchburg, WI, USA). Experiments were carried out in triplicates.

#### 3.4.3 EqUVEC cell migration

EqUVECs (1 × 10^5^ cells/well) were cultured onto a 12-well plate. Upon confluency, a scratch was introduced with a sterile 1,000 μL tip. After removing cell debris by washing with PBS, the scratch filling was documented using a Leica DMi8 inverted microscope (Leica Microsystems GmbH, Wetzlar, Germany) with fixed X and Y positions (4x magnification) for at least three positions/well at defined intervals (0 h, 24 h, 36 h, 48 h, 56 h, and 72 h). Wounded area was analyzed using the Wound Healing Size Tool plugin for ImageJ/Fiji^®^ (39). Experiments were carried out in triplicates.

#### 3.4.4 EqUVEC tube formation

In order to investigate the ability of the cells to form capillary-like structures, tube formation assays were carried out. Ibidi *μ*-angiogenesis slide (Ibidi, Munich, Germany) were coated with 10 μL Matrigel (Thermofisher Science, Carlsbad, California, United States). After polymerization at 37°C for 30 min, the cells were harvested and 1 × 10^4^, 1.5 × 10^4^ and 2 × 10^4^ EqUVECs were seeded. Angiogenesis slides were allowed to incubate at 37°C in 5% CO^2^ humidified ambient oxygen conditions for 30 min. Digital images of the whole wells were captured using a 10x phase contrast magnification on a Leica DMi8 inverted microscope (Leica Microsystems GmbH, Wetzlar, Germany) at 1–3 h-intervals for up to 14 h. Using the Angiogenesis Analyzer plugin for ImageJ/Fiji^®^ number of tubes, number of junctions, and total length of branches and segments were quantified (40).

#### 3.4.5 ELISA for EqUVEC VEGF expression

EqUVECs (3 × 10^4^/well) were cultured onto a 48-well plate. Cell culture supernatants were collected at 24 h-intervals (0 h, 24 h, 48 h, and 72 h) and analyzed for VEGF concentrations using a commercially available, species-specific ELISA kit (Equine VEGF-A ELISA Kit, Invitrogen, Waltham, Massachusetts, United States) according to the manufacturer’s protocol. Standards and samples were added to wells and incubated for 2.5 h at room temperature, followed by an incubation with biotinylated equine VEGF-A for 1 h at room temperature whilst being gently shaken. Streptavidin HRP (1:200) was added to each well and peroxidase activity was determined by incubation with 100 μL peroxidase substrate solution 3,3′,5,5′-tetramethylbenzidine (TMB). Color development was stopped after 30 min and absorbance at 450 nm was quantified using a microplate reader (Promega Corporation, Fitchburg, WI, United States). The standard curve was established by serial dilutions of VEGF-A with a linear range between 1.95 pg./mL and 62.5 pg./mL. Experiments were carried out in triplicates.

## 4 Results

### 4.1 Isolation procedure and culture

**Figure 2:**
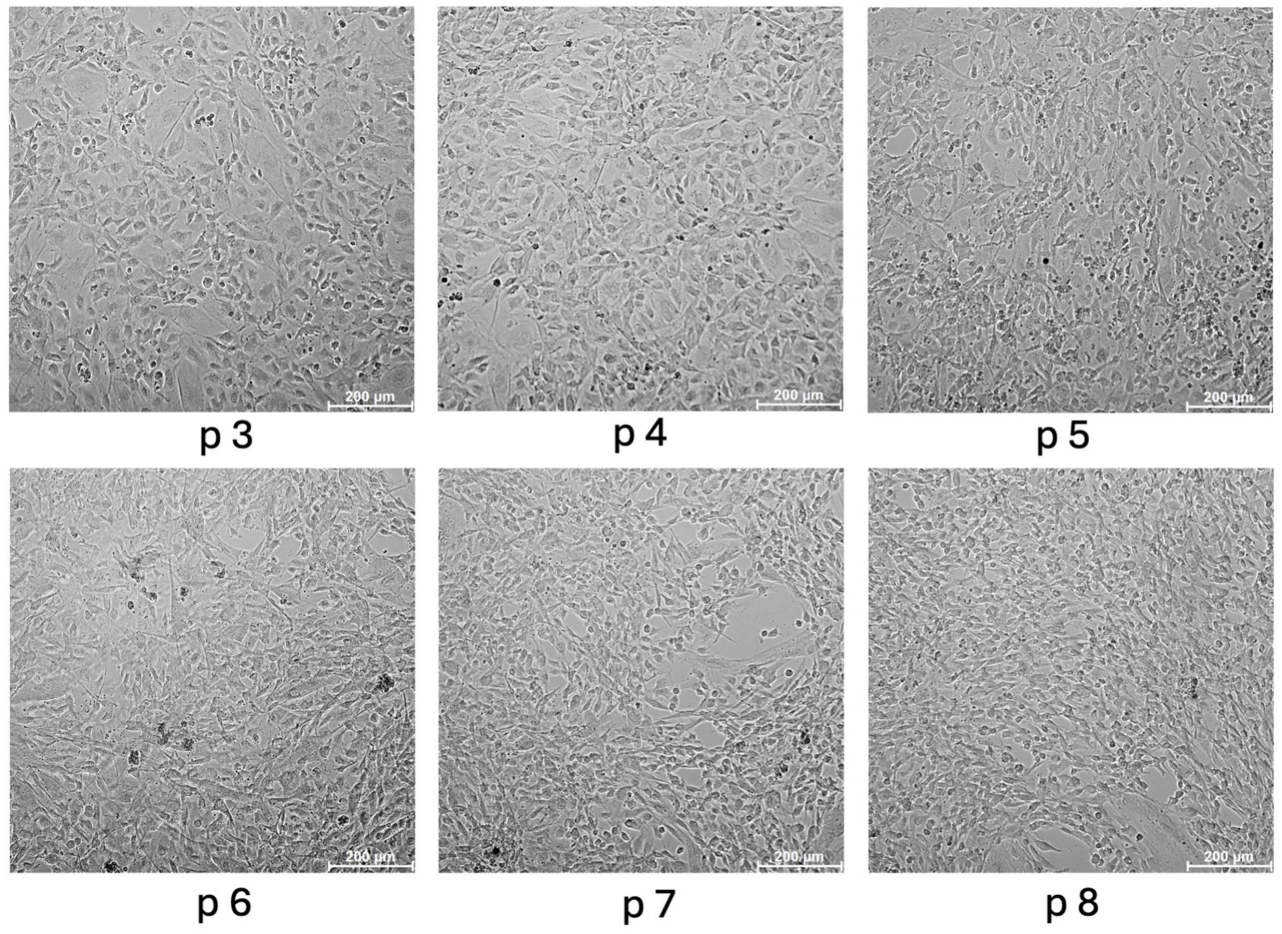
Morphology of equine umbilical vein endothelial cells (EqUVECs). EqUVECs of passage (p) 3 were cultured and after reaching confluency expanded by passaging. Cells were monitored up to passage 8 and photographed by phase contrast microscopy. Scale bars represent 200 μm.

The cells were successfully isolated and cultured in all specimens. Umbilical cords could be sampled quickly and inexpensively and without the need for horses to be euthanized as this is a by-product of natural deliveries. Furthermore, sampling of the cords could be roughly planned, as the mares were hospitalized for birth monitoring. Isolation was performed in one vein per horse with approx. Ten–20 cm length without pooling. Per vein 2.78–7.25 × 10^6^ cells with a viability of 70–82.3% could be isolated (NucleoCounter^®^NC-250, ChemoMetec, Allerod, Denmark). EqUVECs formed a homogeneous monolayer in approximately 1 week, whereas HUVECs showed confluency within 3–5 days. As the primary culture grew and expanded, human and equine cells acquired their typical endothelial shape with long and polygonal cells with a prominent oval nucleus in the cell center. Over passages 3 to 8, EqUVECs exhibited slight morphological changes. Initially, at passage 3, the cells displayed a uniform cobblestone-like appearance typical of endothelial cells. However, with each subsequent passage, cells became increasingly elongated and heterogeneous in shape. By passage 8, the cells showed a more spindle-shaped appearance, indicating potential senescence or phenotypic shift due to extended passaging (Figure 2).

Care must be taken to discard umbilical cords from diseased horses as well as damaged cords. In preliminary experiments one sample was obtained from a foal of a 5-year-old Noriker mare which suffered from inflammatory processes prior to foaling (placentitis). The isolated cells demonstrated the poorest proliferation and survival rate compared to primary cultures of healthy foals, taking 2–3 times longer for monolayer formation. In this period, a significant number of cells perished, especially within the initial days. However, once a monolayer was successfully established and after the subsequent expansion by passaging, their performance in diverse assays (viability, proliferation and migration assay) closely resembled that of cell lines derived from healthy animals (data not shown).

### 4.2 EqUVEC identification and characterization

#### 4.2.1 Morphological characterization of EqUVECs using TEM

**Figure 3:**
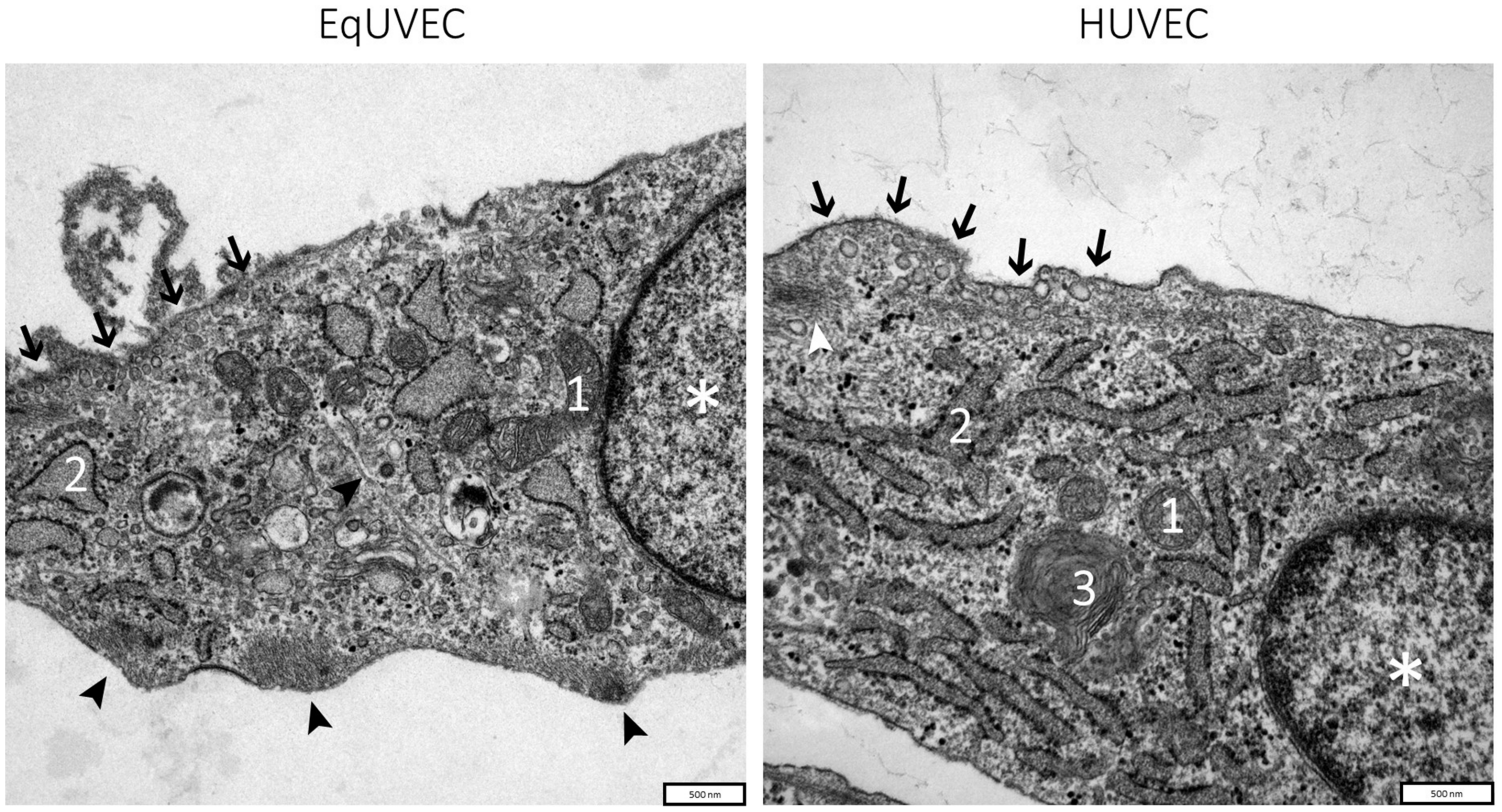
TEM images of equine and human umbilical vein endothelial cells. Overview on cell morphology with nuclei indicated by (*), caveolae on the apical cell domain (➔), cytoplasm with cell organelles like mitochondria (1), rough endoplasmic reticulum with numerous ribosomes on cisternae surface (2), Golgi complexes (3), and agglomerations of the cytoskeletal filaments at adhesion “buds” (➤). Scale bar is 500 nm.

**Figure 4:**
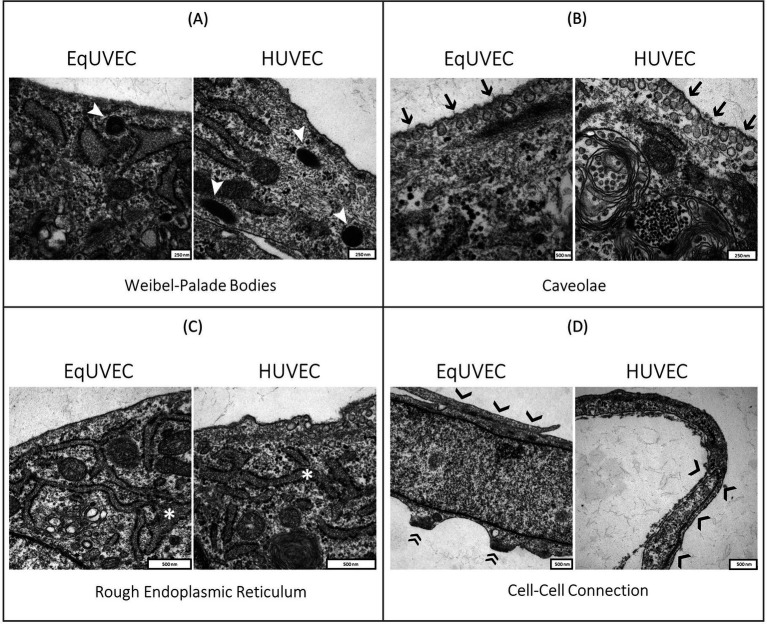
TEM images of equine and human umbilical vein endothelial cells. Close-up of cell morphology with focus on **(A)** Weibel-Palade bodies (➤), **(B)** caveolae on the apical cell domain (➔), **(C)** rough endoplasmic reticulum (*) and **(D)** cell–cell connections formed by desmosomes (❯) and adhesion “buds” (EqUVEC) (»). Scale bar is between 250 and 500 nm.

EqUVECs observed by TEM were homogeneous cells with prominent nuclei rich of euchromatin (Figure 3). EqUVECs rarely contained oval rod-shaped bodies, described previously by Weibel and Palade (WPB) (41, 42), whereas they could be found regularly in the cytoplasm of HUVECs (Figure 4A). In EqUVECs a basement membrane could only be found in some areas, but the cells showed apical-basal polarity with caveolae appearing mainly on the apical cell pole and adherence structures at the basal domain (Figure 4B). The perinuclear region was found to be rich in rough endoplasmic reticulum, mitochondria with tubular shapes, Golgi complexes, pinocytic vesicles and clusters of free ribosomes in human and equine cells (Figures 3, 4C). Furthermore, the oval nuclei presented with a fine granular pattern abundant of euchromatin with prominent nucleoli and enveloped with clearly visible bilayer membranes (nuclear membrane) (Figure 3). TEM observations revealed that cell–cell contacts were created by spot-like adhesions of desmosomes present in overlapping areas of neighboring cells in both cell types. Equine cells formed adhesion “buds” at the basal domain (most likely hemidesmosomes) supported by cytoskeletal elements, which could not be found in HUVECs (Figure 4D). Bundles of cytoskeletal filaments were located throughout the cytoplasm and within the complex interdigitation (EqUVEC) in the peripheral cytoplasm (Figure 3).

#### 4.2.2 Identification of EqUVECs using Immunohistochemical staining

**Figure 5:**
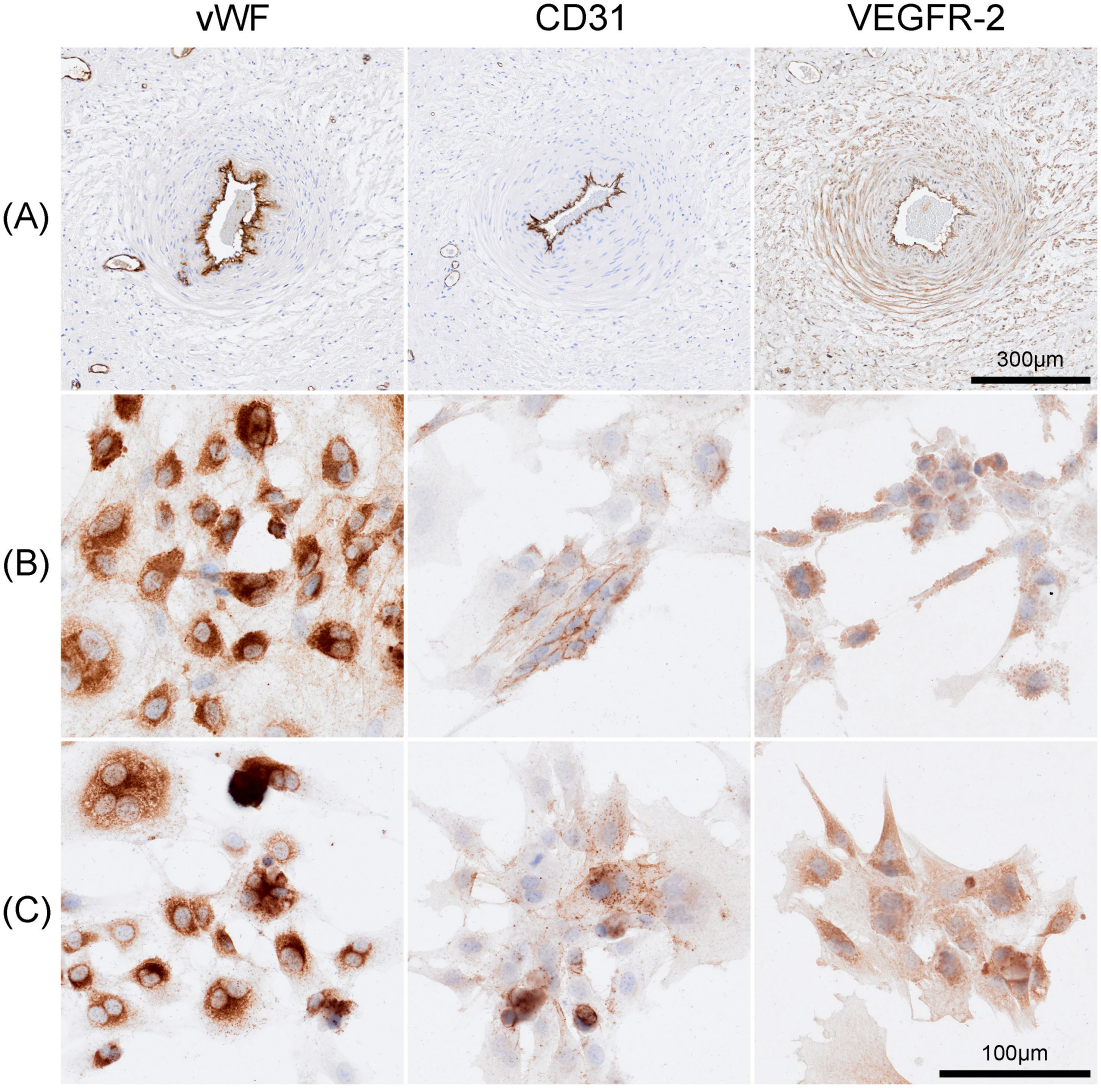
Immunohistochemical staining of Equine umbilical veins and cultured cells. **(A)** Endothelial cells lining the umbilical vein stained strongly positive for anti-von Willebrand factor (vWF), anti-CD-31 and anti-VEGFR-2. Similarly, cells of passage 4 **(B)**, passage7 **(C)** exhibited strong positive staining for these markers. The vWF staining was intense and predominantly cytoplasmic, CD31 expression was primarily localized to the cell membrane, and VEGFR2 showed moderate staining with a heterogeneous pattern observed on both the cell membrane and within the cytoplasm. Scale bar is 100 μm.

Endothelial cells were identified by immunohistochemical staining of vWF, CD-31 and VEGFR-2. Cells showed an intense staining for vWF predominantly localized within the cytoplasm. A heterogeneous staining pattern, with areas showing more concentrated staining, likely reflecting the intracellular processing of vWF, was detected. Cultivated cells stained moderately intense for CD31 and were primarily localized on the cell membrane. The VEGFR2 staining displayed moderate intensity, with varying degrees of staining intensity across different cells mainly localized on the cell membrane but also showing presence within the cytoplasm (Figure 5). Negative controls are shown in Supplementary Figure S1.

### 4.3 Examples of application: performance in different angiogenesis assays

The cell lines were subjected to various assays associated with angiogenesis and their performance was investigated.

#### 4.3.1 EqUVEC cell viability and proliferation

**Figure 6:**
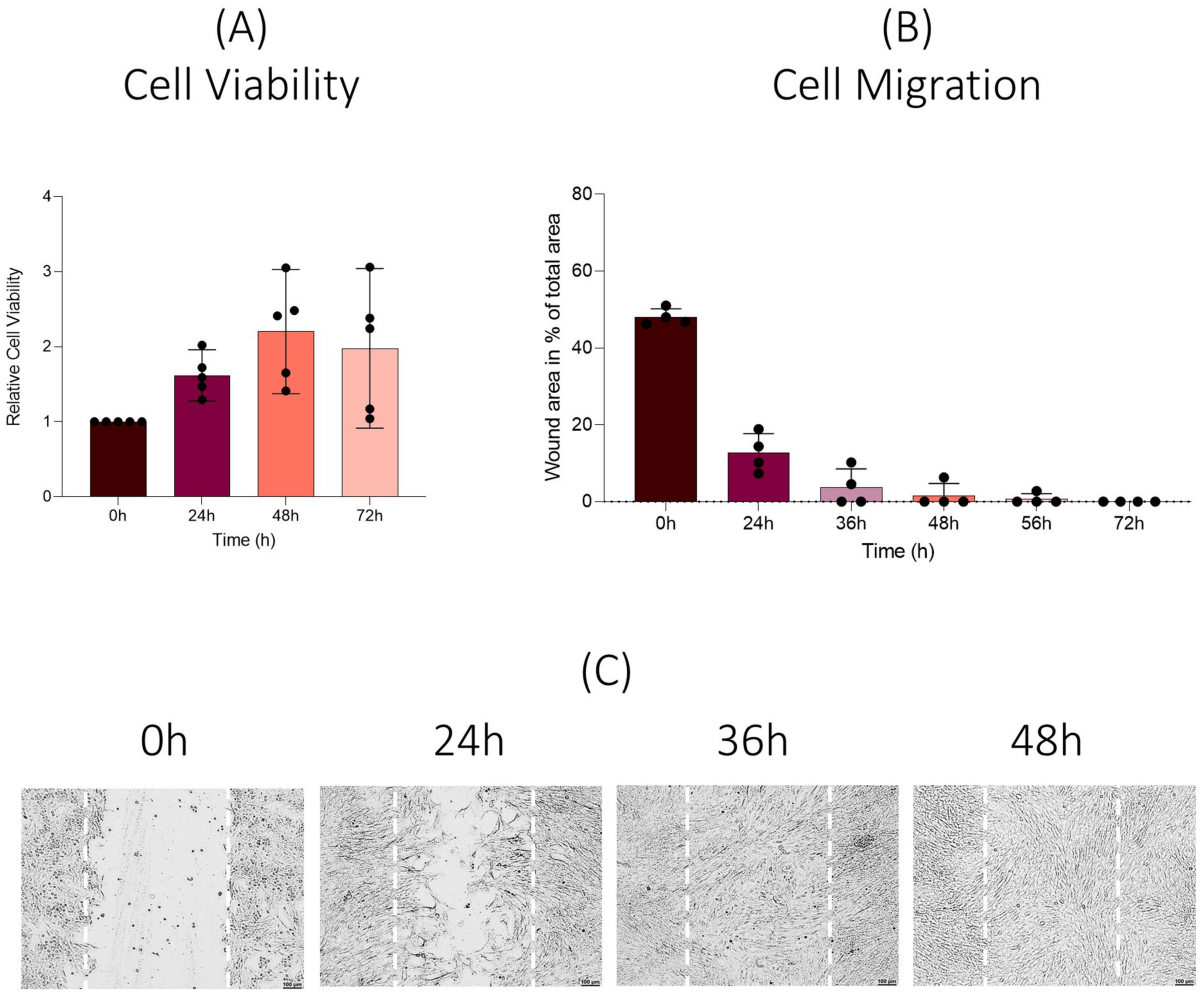
Viability and migration assay. Results are shown as means (*n* = 20). **(A)** Change in cell viability compared to baseline values over a 72 h period (real-time assay). **(B)** Results are shown as means (*n* = 12). Percentage of wounded area at 0 h, 24 h, 36 h, 48 h, 56 h, and 72 h post scratch is shown. **(C)** Representative images after scratch wound.

When analyzed over a 72 h culture period no decrease in relative cell viability was observed throughout the time course (*n* = 20, 24 h = 1.62, 48 h = 2.20, 72 h = 2.05). However, studied cell lines showed an increase in dispersion over the time course with standard deviations of 0.28 (CI: 1.50–1.74) at 24 h, 0.63 (CI: 1.92–2.47) at 48 h and 0.82 (CI: 2.19–3.35) at 72 h (Figure 6A). To determine viable cells in proliferation an end-point assay was carried out. Measured absorbance of the cell lines was 0.92 ± 0.16 (CI: 0.77–0.99) after 72 h.

#### 4.3.2 EqUVEC cell migration

EqUVEC cell migration was assessed by observing their capacity of scratch wound closure. Cells were monitored over 72 h (0 h, 24 h, 36 h, 48 h, 56 h, and 72 h). The migrated cells reduced the wounded area from 48.33% ± 2.89 (*n* = 12; CI: 46.69–49.96) to 12.40% ± 5.27 (*n* = 12, CI: 9.42–15.38) after 24 h and 3.69% ± 4.56 (*n* = 12, CI: 1.11–6.27) after 36 h. After 48 h and 56 h the cell lines showed a further decrease to 1.57% ± 3.02 (*n* = 12, CI: 0–3.28) and 0.69% ± 1.64 (*n* = 12, CI: 0–1.62), respectively. The gap was refilled within 72 h in all cell lines. Cell migration resulted in a confluent monolayer in 50% of the cells within 36 h, in 75% within 48 h and in 83% within 56 h after wound initiation (Figure 6B). Representative images are shown in Figure 6C; images of all time points and cell lines are shown in Supplementary Figure S2.

#### 4.3.3 EqUVEC tube formation

**Figure 7:**
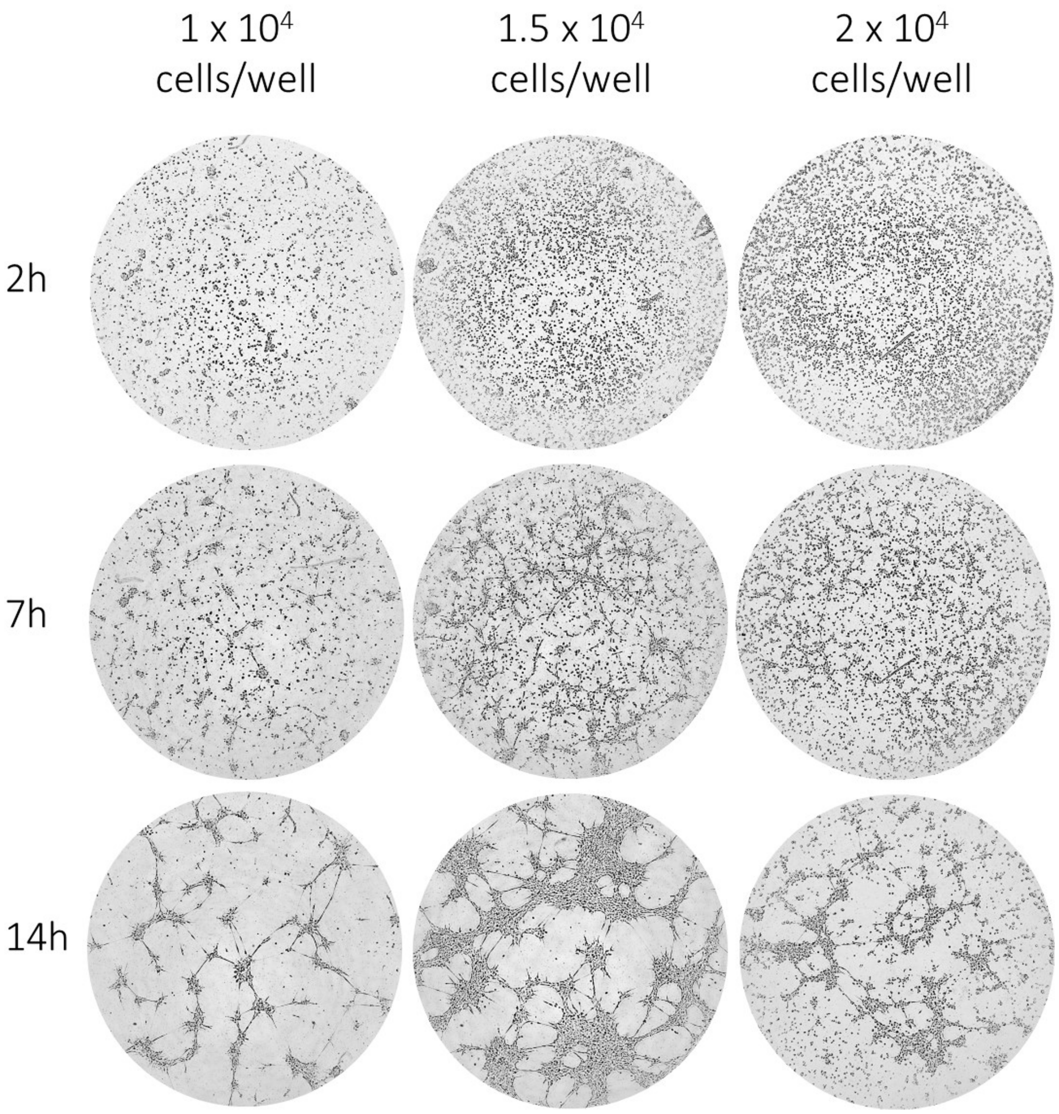
EqUVEC tubular network formation in *in vitro* angiogenesis assay. EqUVEC cells seeded with 1 × 10^4^, 1.5 × 10^4^, and 2 × 10^4^ cells/well growing on Matrigel overnight. Representative images of EqUVECs after 0 h, 7 h, and 14 h of incubation.

Descriptive tube formation assays were carried out in order to assess optimal seeding densities for *μ*-slide angiogenesis assays. Observing a 14 h culture period, EqUVECs formed capillary-like structures when seeded in densities as high as 1 × 10^4^ cells and 1.5 × 10^4^ cells per well, although more pronounced when seeded with 1.5 × 10^4^ cells/well. Higher seeding densities (2 × 10^4^ cells/well) resulted in clumping and cell death. When analyzed (1.5 × 10^4^ cells/well), this resulted in an increase of tubes from 28 (0 h) to 102 (14 h) tubes (+7.63 tubes/h). Similarly, junctions increased from 170 (0 h) to 452 (14 h) junctions (+25.68 junctions/h) and total length of segments and branches from 39,537 (0 h) to 49,888 (14 h) (+855.11 increase in total length/h). Representative images after 2 h, 7 h and 14 h of incubation are shown in Figure 7.

#### 4.3.4 ELISA for EqUVEC VEGF expression

**Figure 8:**
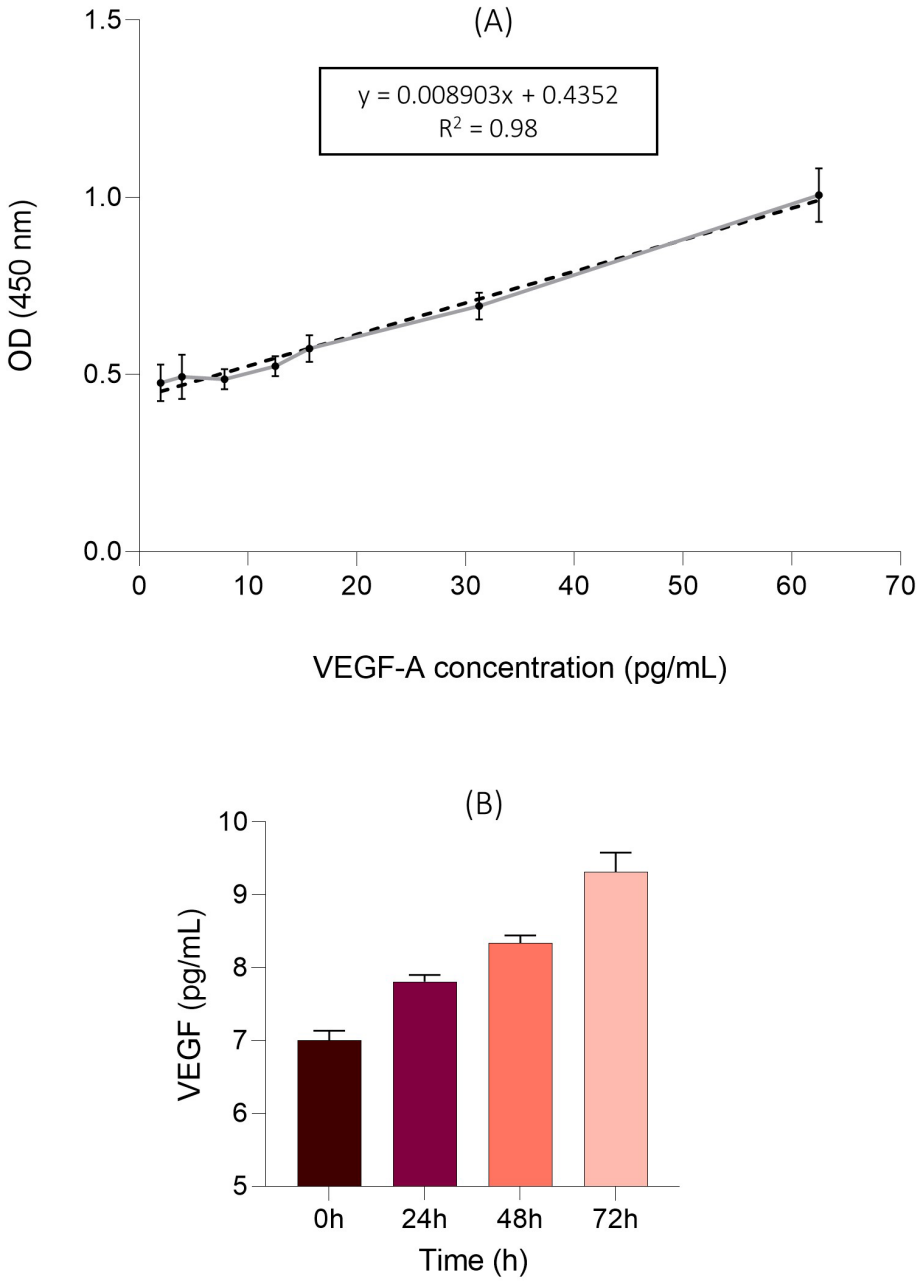
VEGF levels in the supernatants of the EqUVEC cell culture. **(A)** An ELISA standard curve was established to detect 1.95 pg./mL to 62.5 pg./mL of VEGF-A as a reference. The standard curve was generated (*n* = 3) and data points are presented as means with 95% CI. The dotted line rep-resents the best fit determined by linear curve fitting (r^2^ = 0.98). OD = Optical Density. **(B)** Absolute VEGF expression after 0 h, 24 h, 48 h, and 72 h of incubation. Results are shown as means with 95% CI (*n* = 3).

The VEGF-levels in cell culture supernatants were investigated. A standard curve was established using VEGF-A concentrations between 1.95 pg./mL and 62.5 pg./mL. The detected range resulted in a linear relationship (Figure 8A). An increase of VEGF expression was observed over 72 h at all time points. EqUVECs showed VEGF levels of 7.00 pg./mL ± 0.13 (*n* = 3, CI: 6.86–7.15) when seeded. After 24 h and 48 h levels increased to 7.81 pg./mL ± 0.09 (*n* = 3, CI: 7.71–7.91) and 8.34 pg./mL ± 0.11 (*n* = 3, CI: 8.22–8.46). Highest VEGF-level was measured with 9.32 pg./mL ± 0.26 (*n* = 3, CI: 9.03–9.61) after 72 h (Figure 8B).

## 5 Discussion

This study presents a simple protocol for the isolation and culture of equine umbilical vein endothelial cells (EqUVECs), providing a method suitable for application in laboratories with standard equipment. EqUVECs were isolated from the umbilical vein, cultured *in-vitro*, characterized morphologically and immunohistochemically, and evaluated for their performance in various *in-vitro* assays. Documentation on the isolation procedure and utilization of equine ECs from different sources is scarce, with sporadic reports lacking detailed descriptions of the isolation procedures on ECs derived from equine umbilical veins (22–25, 43–51). This protocol was extrapolated from the original description by Baudin et al. (30) for HUVECs.

While developing an isolation protocol it is important to impede culture contamination with foreign cells such as fibrocytes or muscle cells. To promote the selective growth of ECs, non-adherent cells are regularly flushed away after seeding. To further accelerate adhesion of ECs to the plate compared to non-ECs, pre-coated plates could be used (52). While EqUVECs in this study were investigated on uncoated plates without notable contamination, the possible benefits of using matrices like gelatine or fibronectin demand further investigation (52–55).

Choosing an optimal medium is another critical aspect of establishing an endothelial cell culture. Fetal calf serum (FCS) was used as the primary medium supplement due to its high content of adhesion proteins, immunoglobulins, transcription factors, nutrients, and growth factors, among other vital components (56). Fetal horse serum (FHS) is also suitable for equine endothelial cell cultures and specific applications May benefit from species-specific serums, as evidenced in the cultivation of porcine intestinal cells (48, 57). However, data provided by Dietze et al. (22) indicate that FHS did not increase equine EC proliferation. The possible superiority of FHS for EqUVEC culture was not assessed and needs further investigations (22).

When culturing ECs, it is recommended to use specific culture media containing not only amino acids, vitamins, carbohydrates and salts, but also a variety of growth factors in addition to those found in fetal bovine sera (35). This approach is crucial for the optimal growth and maintenance of ECs derived from various species, including humans, rhesus macaques, and ovines (58–60). Commonly used growth factors known for their high mitogenic, and selective properties include fibroblast growth factors (FGFs), platelet-derived endothelial growth factors (PD-EGF) and vascular endothelial growth factors (VEGF) (16, 61, 62). However, this study opted to not use supplementary growth factors, considering their potential influence on protein synthesis, intracellular exchange and impact as a compensatory factor (63–66). Despite not supplementing the media, we were able to successfully isolate and cultivate EqUVECs demonstrating the adaptability of the cells to grow and proliferate in basic culture conditions. Several published protocols described the successful isolation and characterization of endothelial cells from umbilical cords of species such as bovines and swine using non-supplemented media (67, 68). These studies highlight that endothelial cells from these species can be effectively cultured and maintained even without additional growth factors.

Cultures needed to be checked for contaminations, such as bacteria, fungi and foreign cells, regularly. Most bacterial and fungal contaminations can be identified by light microscopy; however, mycoplasma remains usually undetected. Therefore, all cell cultures underwent mycoplasma DNA detection using PCR prior to conducting further experiments. To prevent contamination of foreign cells, non-adherent cells were regularly flushed away after seeding. Most markers used to identify foreign cells, such as fibroblasts and smooth muscle cells, are also expressed in endothelial cells, making them insufficient for foreign cell identification (69). However, these cells display characteristic features, such as fibroblasts often exhibiting a spindle-shaped morphology and forming a characteristic mesh-like pattern, while smooth muscle cells are typically elongated and exhibit a “hill and valley” growth pattern. These distinct features aid in the identification of foreign cells in cultures (70, 71).

Identification of endothelial cells depends on various factors, including cell morphology, the presence of marker organelles and immunolabeling with protein markers. In this study, EqUVECs exhibited characteristics similar to those described for HUVECs, although Weibel-Palade bodies (WPBs) were rarely observed. Immunohistochemical staining confirmed the presence of cell markers, including vWF, CD31, and VEGFR-2, supporting the endothelial character of the cells. The cellular identity of ECs can be ascertained through cell morphology, the presence of endothelial marker organelles and immunolabeling with specific markers (21). In this study, EqUVECs exhibited characteristics similar to those of HUVECs initially characterized by Jaffe in 1973, although Weibel-Palade bodies (WPBs) were rarely observed (72). Weibel-Palade bodies are oval-or rod-shaped secretory organelles specifically found in ECs of numerous species and found to release von Willebrand factor (vWF) (11, 21, 73–77). The discrepancy in observing Weibel-Palade bodies could be explained by an overall difficulty in locating these organelles, as well as a low observation frequency described by Jaffe (72).

While ECs exhibit numerous morphological characteristics, relying solely on microscopic examination is insufficient for their identification. Endothelial cells can be identified by immunohistochemical staining; however, due to their diversity, there is no universal marker (21). Despite forming a single-layered lining in blood vessels and the heart, ECs display notable variations in function, morphology, and antigen expression (78). Different cell markers can be identified in the cell’s cytoplasm and membrane. In horses, among other species, the von-Willebrand-factor is seen as a specific endothelial marker (79, 80). In this study, positive vWF labelling affirmed the endothelial-specific character of the cells. To ensure consistency, cells up to passage 5 were used in the experiments, since fading or loss of vWF labelling has been reported in advanced cell culture passages of other endothelial cultures, including equine ECs (81). This decline is attributed to an aging mechanism coupled with spontaneous apoptosis, as evidenced by decreased angiotensin I-converting enzyme (ACE) and prostacyclin synthesis in advanced cell culture passages (82–85).

Furthermore, EqUVEC stained positive for CD31 and VEGFR-2. CD31, a glycoprotein belonging to the immunoglobulin (Ig) superfamily, serving as a universal endothelial marker situated in the EC membrane near the intercellular junction (86). While VEGFR-2 is being highly expressed by endothelial cells, it is also present in various other cell types such as luminal and glandular epithelium of the endometrium, trophoblasts and the fetal epithelium (87, 88). Despite not being a specific endothelial marker, investigating VEGFR-2 expression in EqUVECs is crucial given its potential as an *in-vitro* model for angiogenesis in horses. Interactions between VEGF-A and its receptors (VEGFR 1, 2, and 3) are pivotal in angiogenesis. In horses, numerous physiological and pathological conditions are influenced by the VEGF-VEGFR axis, including embryonic vascular development, wound healing, chronic inflammatory diseases and ocular neovascular diseases (9, 11, 89, 90). Additionally, the VEGF–VEGFR-2 signaling pathway is extensively studied and holds significant importance as a promoter of pathological vascularization in tumor development and progression (91). To assess VEGF-A expression in EqUVECs, an ELISA approach was chosen to measure VEGF levels in the cell culture supernatants. An increase in VEGF levels by approximately 33% was observed, with the highest VEGF concentration reaching 9.32 pg./mL ± 0.26 after 72 h. Sadick et al. (33) reported a 64% increase in VEGF expression over 72 h, with the highest VEGF level reaching 41.54 mg/mL in humans. This disparity suggests limited comparability of human and equine VEGF expression in endothelial cells, underscoring the necessity for further investigations. In a previous study, we demonstrated, that vascular endothelial growth factor inhibitors efficiently inhibit VEGF-associated cellular processes and lead to a decrease in VEGF expression (38). Future investigations should focus on evaluating the specific effects of VEGF on these cells. Stimulating cell cultures with growth factors could provide deeper insights into signal pathways and cellular mechanisms involved in these processes.

*In-vitro* models, while being unable to replicate all aspects of physiological angiogenesis, can successfully simulate *in-vivo* conditions, although certain limitations, like cellular heterogeneity and disparities in growth rates between *in vitro* and *in vivo* settings have to be considered (39). Standardized preparations, media, and cell culture passages are essential to address cellular heterogeneity. Gender differences, including variations in cell proliferation, migration and protein expression, as observed in HUVECs, should also be taken into account (92). The current study investigated umbilical cords of three male and two female foals, which showed generally homogeneous performance. However, due to the small sample size, robust conclusion cannot be drawn, and future research should investigate potential sex-based differences.

Additionally, conducting a set of assays targeting distinct stages of angiogenesis is crucial for obtaining comprehensive insights into the process (3). This study assessed the performance of EqUVECs in viability, proliferation, migration and tube formation assays. Metabolic assays, readily accessible and requiring minimal handling and equipment, were used to investigate cell proliferation. The most well-known assay is the MTT assay (3-(4,5-dimethylthiazol-2-yl)-2,5-diphenyltetrazolium bromide), which induces a color change upon reduction of the tetrazolium compound by metabolically active cells. Furthermore, proliferation and viability assays serve as valuable tools for chemo-sensitivity and cytotoxicity testing of drugs (93, 94). EC migration is a crucial aspect of angiogenesis, representing an early stage in the angiogenic cascade (95–97). It is characterized by autonomous cell motility and collective migration with groups of cells coordinating movements towards a chemotactic gradient, thus establishing a hierarchical structure with leader and follower cells (98). Investigating the molecular mechanisms behind EC migration is essential for a comprehensive understanding and future therapeutic intervention, like inhibition of angiogenesis in tumors or stimulation of vessel formation during wound healing (3). One of the basic tools for cell migration assessment is the cell culture wound closure assay. Various closure times have been observed in equine ECs derived from different vessels, spanning from 15 h to 40 h (78). This emphasizes the importance of determining the migration rate in ECs derived from umbilical veins and cultured in different settings. EqUVECs reformed a monolayer within an average of 47 h with consistently similar closure times observed across different cell lines. Interestingly, achieving a uniform scratch proved to be considerably more challenging in equine cells compared to HUVECs. This finding was also noted by Rieger et al. (78) in equine ECs obtained from jugular veins, despite being cultured under varied conditions. The tube-formation-assay is another powerful tool reflecting the coordination of various cellular processes such as proliferation, migration and apoptosis (69). Care must be taken when interpreting the tube formation assay, due to the use of a single replicate. However, when seeded at a density of 1.5 × 10^4^ cells/well EqUVECs formed capillary-like structures within 14 h.

Viability-, proliferation-, migration-and tube-formation-assays were found to be applicable in EqUVECs. Future studies should include more advanced angiogenesis assays, such as 3D spheres, to evaluate processes like VEGF-dependent sprout and lumen formation in extracellular matrices. These approaches would provide deeper insights into equine angiogenesis.

## 6 Conclusion

In conclusion, this study aimed to address the need for suitable *in-vitro* models for angiogenesis in equine research by isolating and characterizing equine umbilical vein endothelial cells (EqUVECs). The results demonstrate that EqUVECs can serve as a valuable model for studying endothelial function and angiogenesis, similar to the well-established human umbilical vein endothelial cell (HUVEC) model. This achievement highlights the potential of EqUVECs for investigating angiogenesis-related processes and therapeutic interventions in horses. Nevertheless, future studies should aim to investigate specific properties and behaviors of EqUVECs in experimental setups.

## Data Availability

The original contributions presented in the study are included in the article/Supplementary material, further inquiries can be directed to the corresponding author.

## Supplementary material

SUPPLEMENTARY FIGURE 1 Negative controls of immunohistochemical staining for anti-rabbit secondary antibody (VEGFR2) and anti-mouse secondary antibody (CD31 and vWF). Scale bar is 100 µm.
