# Supplementary figures and images for: Isolation-protocol, characterization, and in-vitro performance of equine umbilical vein endothelial cells
Source: Front Vet Sci. 2024 Oct 1;11:1421946. doi: 10.3389/fvets.2024.1421946 (PMC11473255; doi:10.3389/fvets.2024.1421946)

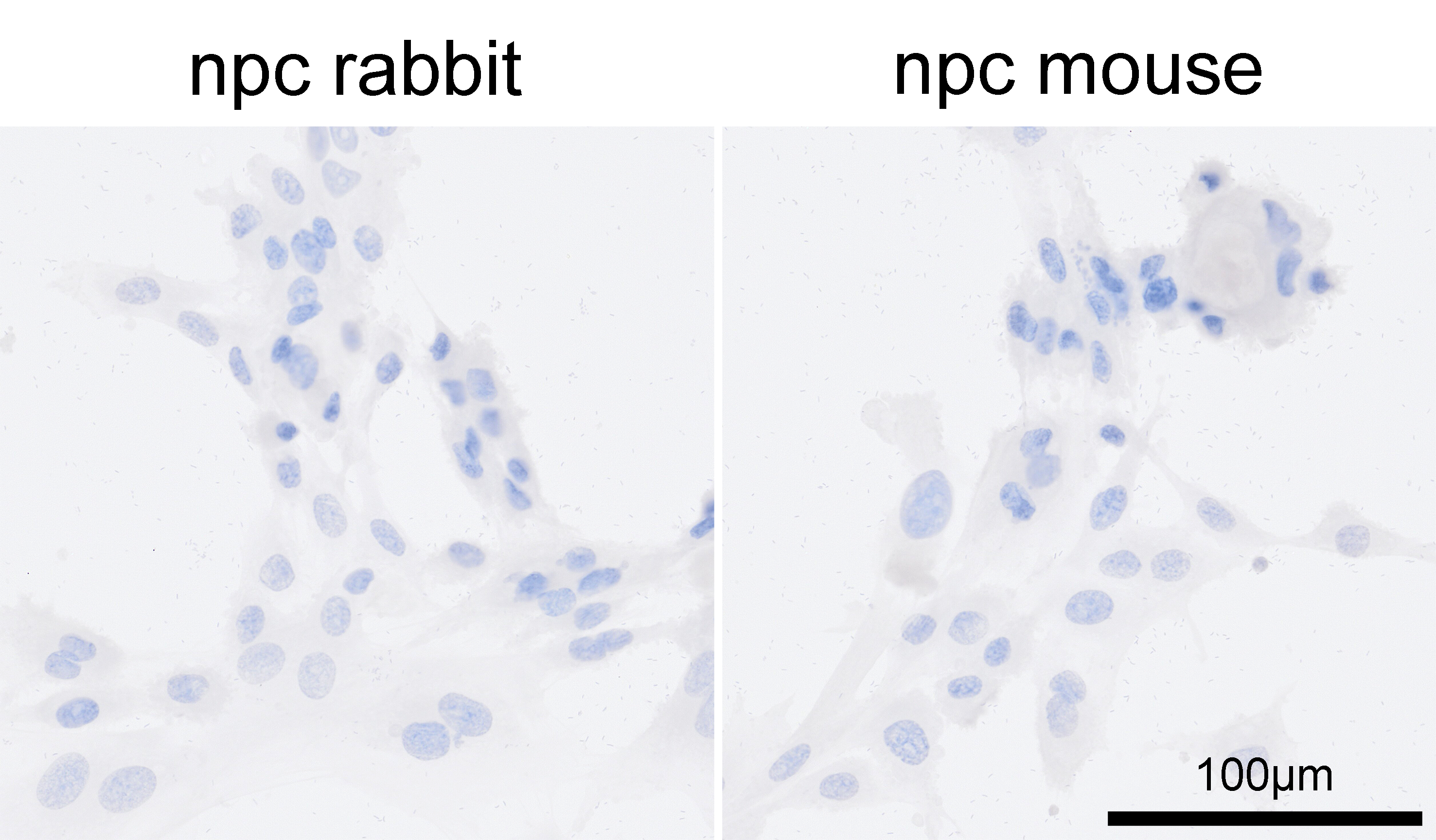

Supplement: SUPPLEMENTARY FIGURE 1 — Negative controls of immunohistochemical staining for anti-rabbit secondary antibody (VEGFR2) and anti-mouse secondary antibody (CD31 and vWF). Scale bar is 100 µm. [file Image_1.jpeg]

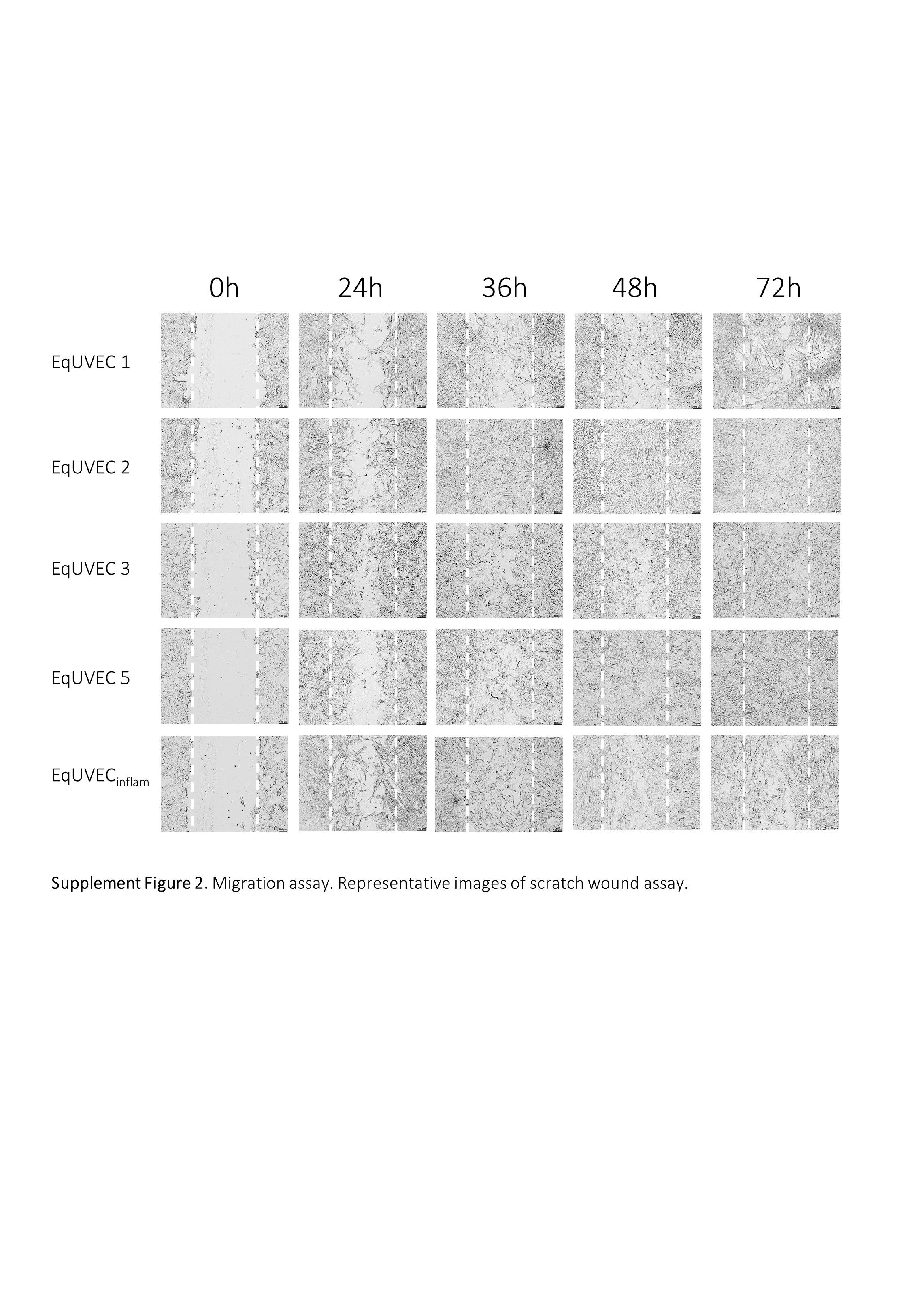

Supplement: Supplementary file 2 [file Image_2.jpeg]
